# Systematic review of clinical practice guidelines for adults with fractures: identification of best evidence for rehabilitation to develop the WHO’s Package of Interventions for Rehabilitation

**DOI:** 10.1186/s10195-020-00560-w

**Published:** 2020-11-14

**Authors:** Francesca Gimigliano, Sara Liguori, Antimo Moretti, Giuseppe Toro, Alexandra Rauch, Stefano Negrini, on behalf of other members of the Technical Working Group, Claudio Curci, Michele Patrini, Livia Peschi, Sanaz Pournajaf, Maria Sgarbanti, Giovanni Iolascon

**Affiliations:** 1 Department of Mental and Physical Health and Preventive Medicine, University of Campania “Luigi Vanvitelli”, Largo Madonna delle Grazie n. 1, 80138 Naples, Italy; 2 Department of Medical and Surgical Specialties and Dentistry, University of Campania “Luigi Vanvitelli”, Via De Crecchio n.4, 80138 Naples, Italy; 3 Rehabilitation Programme World Health Organization, Avenue Appia 20,, 1211 Geneva 27, Switzerland; 4 IRCCS Istituto Ortopedico Galeazzi, Milan, Italy; 5 Department of Biomedical, Surgical, and Dental Sciences, University La Statale, Milan, Italy

**Keywords:** Fractures, Adult population, Clinical practice guidelines, Rehabilitation, World Health Organization

## Abstract

**Background:**

The identification of existing rehabilitation interventions and related evidence represents a crucial step along the development of the World Health Organization’s (WHO) Package of Interventions for Rehabilitation (PIR). The methods for such identification have been developed by the WHO Rehabilitation Programme and Cochrane Rehabilitation under the guidance of the WHO’s Guideline Review Committee secretariat. The aim of this paper is to report on the results of the systematic search for clinical practice guidelines (CPGs) relevant to the rehabilitation of adults with fractures and to present the current state of evidence available from the identified CPGs.

**Methods:**

This paper is part of the Best Evidence for Rehabilitation (be4rehab) series, developed according to the methodology presented in the World Health Organization’s (WHO) Package of Interventions for Rehabilitation (PIR) introductory paper. It is a systematic review of existing CPGs on fractures in adult population published from 2009 to 2019.

**Results:**

We identified 23 relevant CPGs after title and abstract screening. According to inclusion/exclusion criteria, we selected 13 CPGs. After checking for quality, publication time, multiprofessionality, and comprehensiveness, we finally included five CPGs dealing with rehabilitative management of fractures in adult population, two CPGs addressing treatment of distal radius fracture and three the treatment of femoral/hip fracture.

**Conclusion:**

The selected CPGs on management of distal radius and femoral/hip fracture include few recommendations regarding rehabilitation, with overall low to very low quality of evidence and weak/conditional strength of recommendation. Moreover, several gaps in specific rehabilitative topics occur. Further high-quality trials are required to upgrade the quality of the available evidence.

**Level of evidence:**

Level 1.

## Introduction

The WHO has the strategic priority of achieving Universal Health Coverage (UHC), which means that “all people receive quality health services that meet their needs without being exposed to financial hardship in paying for the services” [1]. UHC includes rehabilitation among the services to be provided. As part of the WHO Rehabilitation 2030 call for action [2], the WHO Rehabilitation Programme is developing its Package of Interventions for Rehabilitation (PIR, formerly the Package of Rehabilitation Interventions) to support ministries of health in integrating rehabilitation services into health systems [3].

The development of the PIR takes a stepwise approach [3]. The second step, referred to herein as be4rehab, requires the identification of interventions for rehabilitation and related evidence for the health conditions selected in the first step. The WHO Rehabilitation Programme and Cochrane Rehabilitation developed the corresponding methodology under the guidance of the WHO’s Guideline Review Committee secretariat and are collaborating in conducting this step. Be4rehab includes a series of systematic reviews on clinical practice guidelines (CPGs) for the different health conditions. Interventions and related evidence are identified from these. The identified interventions will be subject to a consensus process before being included in the final PIR, and information related to their provision will be added. All information will undergo a review process before the development of the final version of the PIR.

The worldwide incidence of fractures in the adult population is reported to range between 9.0 and 22.8/1000/year [4]. Hip fractures are the most common fractures in the adult population. Age is the main risk factor for such fractures, and with the global increase of life expectancy, it is estimated that the total number of hip fractures will reach 6.26 million by 2050 [5]. Regarding upper extremity fractures, an incidence of about 67/10,000/year is estimated in the USA, of which distal radius and ulnar fractures account for about 25% [6]. Global incidence rates of distal radius fracture range from 4 to 110/10,000/year [7], representing (after hip) the second most common fracture in patients aged over 65 years.

Rehabilitation following fracture is mandatory to prevent complications, optimize functional recovery, and achieve independence of activities of daily living (ADLs) [8]. In the elderly population, it is important to ensure access to rehabilitation as part of continuity of care to avoid functional and cognitive deterioration [9]. Hip fractures, for example, represent a dramatic event after which only half of patients improve their mobility and 30% will not regain autonomy in ADLs without receiving rehabilitation [10]. Moreover, recovery of independence and functional autonomy after fractures are essential outcomes from the patient perspective [11].

The objective of this paper is to report on the results of the systematic search for CPGs relevant to rehabilitation of adults with fractures, limiting the search to the following sites: humerus, radius, femur/hip, and tibia. The specific objectives are to present the topics of the recommendations and the current state of evidence available from the identified CPGs.

## Methods

**Fig. 1:**
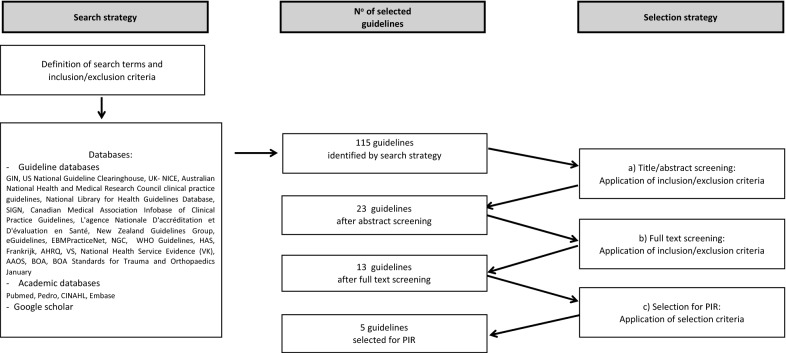
The results of the screening process

This systematic review of CPGs was developed in full compliance with the methodology presented in the introductory PIR paper [3], based on the following stages (Fig. 1):

- Systematic literature search: The following databases were searched for CPGs: PubMed, Pedro, CINAHL, Embase, Google Scholar, Guidelines International Network (GIN), US National Guideline Clearinghouse, UK National Institute for Clinical Excellence (NICE), Australian National Health and Medical Research Council clinical practice guidelines, National Library for Health Guidelines Database (UK), Scottish Intercollegiate Guidelines Network (SIGN), Canadian Medical Association Infobase of Clinical Practice Guidelines, l'Agence nationale d'accréditation et d'évaluation en santé (France), New Zealand Guidelines Group, eGuidelines, EBMPracticeNet, National Guideline Clearinghouse (NGC), WHO Guidelines, Haute Autorité de santé (HAS), France, Agency for Healthcare Research and Quality (AHRQ, US), National Health Service Evidence (UK), American Academy of Orthopaedic Surgeons (AAOS), British Orthopaedic Association (BOA), BOA Standards for Trauma and Orthopaedics January 2012, Société Française de Chirurgie Orthopédique et Traumatologique (SOFCOT), British Society for Surgery of the Hand (BSSH), and National Health Society (NHS). Considering the heterogeneity of the rehabilitation needs after fractures of different bones, we decided to limit the search to long appendicular bones, namely humerus, radius, femur/hip, and tibia. The search strategies are reported in Appendix 1. The search was performed on 27 February 2019, and included all documents from 2009 to 2019 regarding CPGs on fractures in both children/youths and adults, progressing as follows:
- Independent abstract and full-text screening of the retrieved documents by members of the Technical Working Group (TWG);
- Independent quality evaluation of the CPGs using the Appraisal of Guidelines for Research and Evaluation (AGREE II) tool by two members of the TWG [12], with a specific focus on items 7, 8, 12, and 22, for which the average result had to be > 2 (AGREE/4), and items 4, 7, 8, 10, 12, 13, 15, 22, and 23, whose average sum score had to be > 45 (AGREE/9);
- The final selection of a maximum of five CPGs for each age group (children/youths and adults), according to the following criteria: (1) quality, (2) publication time, (3) multiprofessionality, and (4) comprehensiveness. This decision was reached by agreement of the whole group;
- Data extraction using a standardized form, comprising information on the recommendation (type of recommendation, dosage, target group, etc.), the strength of the recommendation, and the quality of the evidence used to inform the recommendation.

No changes were made to the published protocol. The quality check and methodological support for this study were provided by Cochrane Rehabilitation.

The topics addressed by each CPG for the different types of recommendation (service, assessment, and intervention) were extracted. The topics from the first CPG were compared independently by two authors and integrated with those coming from the second. If required, agreement was reached by discussion involving a third author. This process was repeated for all the CPGs until final agreement on the topics was achieved.

## Results

The results of the selection process are reported in Fig. 1. Our TWG identified 23 relevant CPGs on fractures in both children/youths and adults based on title and abstract screening. We excluded eight of them for the following main reasons (Table 1): AGREE/9 score ≤ 45 [13–20]; moreover, in two of them, the absence of possible conflict of interest was not clearly stated [13, 14] and in four the strength of recommendations was not reported [14–17], while six had AGREE/4 score ≤ 2 [13–18]. We then excluded another two documents as one was only a summary [21] and the other was a duplicate of another GPC with a different document title [22].

**Table 1:** Guidelines found and selected, and their assessment against the criteria used to reach the final choice

Guideline	AGREE ratings						Multiprofessional team (Y/N)	Topic	Publication date (Y/N)
	Total	Average of key items							
		7	8	12	22	4, 7, 8, 10, 12, 13, 15, 22, 23			
Included									
AAOS radius [29]	100	7	7	7	7	63	Y	Distal radius fracture	2009
DHA radius [30]	99	7	7	7	7	63	Y	Distal radius fracture	2016
NICE hip [31]	100	7	7	7	7	63	Y	Hip fracture	2011
ANZHFR hip [32]	100	7	7	7	7	63	Y	Hip fracture	2014
AAOS hip [33]	100	7	7	7	7	63	Y	Hip fracture in elderly	2015
Excluded at the final selection									
Lichtman [23]	86	7	7	7	7	61	Y	Distal radius fracture	2011
Qaseem [24]	82	7	6.5	6	6	55.5	N	Osteoporosis and fracture	2017
Dachverband Osteologie e.V. [25]	67	5	5	7	5	49	Y	Osteoporosis	2011
British Society for Surgery of the Hand (BSSH) [26]	75	7	7	7	4	53	Y	Distal radius fracture	2018
Mak et al. [27]	58	7	5.5	6	5.5	46.5	N	Hip fracture in older people	2010
Scottish Intercollegiate Guidelines Network (SIGN) [28]	86	7	7	7	2	52	Y	Hip fracture in older people	2009
Excluded for not respecting the inclusion criteria									
Licthman et al. [13]	33	1	1	1	2	16	N	Distal radius fracture	2011
Tarantino [20]	76	4	2.5	6	6	43	Y	Osteoporosis	2017
Singleton [16]	33	1.5	1	1.5	7	23.5	N	Proximal humeral fracture	2014
Swift [15]	57	1.5	1	2	6	27.5	Y	Hip fracture	2016
Wilson [14]	22	1	1	2	1	13	N	Hip fracture	2013
Sherrington [19]	62	6	4	7	7	44.5	N	Hip fracture	2011
Dehghan [18]	48	1.5	1	5.5	6	33.5	N	Upper and lower extremity fractures	2018
McBrien [17]	14	1	1	1	1	9	N	Hip fracture and clopidogrel	2013

After rating the remaining 13 guidelines, our TWG discussed and then excluded 6 CPGs from the final selection based on one of the following criteria: quality, publication time, multiprofessionality, and comprehensiveness [23–28]. We finally selected five CPGs for adults and the elderly: the American Academy of Orthopaedic Surgeons treatment of distal radius fractures, 2009 (AAOS radius) [29]; the Danish Health Authority national clinical guideline on the treatment of distal radial fractures, 2016 (DHA radius) [30]; the National Clinical Guideline Centre management of hip fracture in adults, 2011 (NICE hip) [31]; the Australian and New Zealand Hip Fracture Registry Steering Group Australian and New Zealand guideline for hip fracture care: improving outcomes in hip fracture management of adults, 2014 (ANZHFR hip) [32]; and the American Academy of Orthopaedic Surgeons management of hip fractures in the elderly, 2014 (AAOS hip) [33] (Table 1).

The median AGREE II rating for the identified CPGs was 75 (14–100), while that of the selected CPGs was 100 (99–100) (Table 1).

Overall, we included two CPGs for treatment of distal radius fracture and three for treatment of femoral/hip fracture. In particular, we extracted 19 recommendations: 11 on service, 2 on assessment, and 6 on intervention. The identified recommendations per type and topic (functional domain) considered by the selected CPGs are summarized in Tables 2 and 3. The quality of evidence was overall low to very low, with weak/conditional strength of recommendation, as reported in Table 4.

**Table 2:** Number of recommendations per type in each guideline

Guideline	No. of recommendations on		
	Service	Assessment	Intervention
AAOS radius [29]	1 (25%)	0 (0%)	3 (75%)
DHA radius [30]	1 (50%)	0 (0%)	1 (50%)
NICE hip [31]	3 (60%)	1 (20%)	1 (20%)
ANZHFR hip [32]	3 (60%)	1 (20%)	1 (20%)
AAOS hip [33]	3 (100%)	0 (0%)	0 (0%)

**Table 3:** Number of recommendations identified per topic (functional domain) and type (service, assessment, and intervention)

Topic	Selected guidelines				
	AAOS radius [29]	DHA radius [30]	NICE hip [31]	ANZHFR hip [32]	AAOS hip [33]
Service recommendations					
Multidisciplinary management	Home exercise program (p. 84, recommendation 21)	No rehabilitation supervised by an occupational therapist or a physiotherapist on a routine basis for patients with uncomplicated cases; offer guidance and practical instruction concerning self-rehabilitation (p. 46, recommendation 7.2)	Physiotherapy assessment and mobilization on day after surgery (p. 134, recommendation 11.2.2) From admission, formal, acute orthogeriatric or orthopedic ward-based hip fracture programme (p. 157, recommendation 12.2.3) Consider early supported discharge as part of the hip fracture programme (p. 171, recommendation 12.4.4) Continued rehabilitation in a community hospital or residential care unit (p. 172, recommendation 12.4.4) Patients admitted from care or nursing homes should not be excluded from rehabilitation programs in the community or hospital (p. 173, recommendation 12.4.4)	Mobilization the day after surgery and physiotherapy assessment (p. 63, recommendation 6.1) From admission, formal, acute orthogeriatric service (p. 69, recommendation 7.1) Consider early supported discharge (p. 74, recommendation 7.2)	Use of an interdisciplinary care program in patients with mild to moderate dementia (p. 262) Supervised occupational and physical therapy across the continuum of care (p. 259) Intensive home physical therapy (p. 260)
Assessment recommendations					
Cognitive assessment			Deliver care that minimizes the patient’s risk of delirium and maximizes their independence (p. 162, recommendation 12.2.3)	Deliver care that minimizes the patient’s risk of delirium and maximizes their independence (p. 73, recommendation 7.1)	
Intervention recommendations					
Therapeutic exercise	Active finger motion exercises (p. 88, recommendation 22) No need for early wrist motion following stable fracture fixation (p. 89, recommendation 23)		Mobilization at least once a day and regular physiotherapy review (p. 137, recommendation 11.3.2)	Mobilization at least once a day and regular physiotherapy review (p. 65, recommendation 6.2)	
Physical agents	Ultrasound and/or ice adjuvant treatment (p. 98, recommendation 27)				
Orthosis and prosthesis		Consider short-term cast or similar immobilizing bandage (< 2 weeks) following insertion of a volar angular stable locking plate rather than long-term cast or similar immobilizing bandage (> 5 weeks) (p. 42, recommendation 6.2)

**Table 4:** Strength of recommendation and quality of evidence in the selected guidelines

Guideline	Body of evidence			Strength of recommendation		
	RCTs, systematic reviews, or metaanalyses*	Clinical studies	Expert opinion	Strong	Intermediate	Weak
AAOS radius [29]	10 (91%)	0 (0%)	1 (9%)	0 (0%)	1 (25%)	3 (75%)
DHA radius [30]	4 (100%)	0 (0%)	0 (0%)	0 (0%)	1 (50%)	1 (50%)
NICE hip [31]	16 (84.2%)	3 (15.8%)	0 (0%)	1 (12.5%)	2 (25%)	5 (62.5%)
ANZHFR hip [32]	18 (95%)	0 (0%)	1 (5%)	1 (20%)	2 (40%)	2 (40%)
AAOS hip [33]	20 (100%)	0 (0%)	0 (0%)	2 (75%)	1 (25%)	0 (0%)

Since the reference scales adopted by each guideline are not directly comparable, we present the recommendations according to two summary three-point Likert scales

* At least one RCT or one systematic review are required to classify in this column

Regarding the recommendations on rehabilitation following distal radius fracture, the AAOS radius CPG recommends a home exercise program as an option for patients’ functional recovery (strength of recommendation weak/conditional—“limited”; quality of evidence moderate—“level II”) and the DHA radius CPG recommends providing only practical instructions on self-rehabilitation after distal radius fracture in uncomplicated cases (strength of recommendation expert opinion—“good practice”; quality of evidence very low) [29, 30]. In relation to interventions, the AAOS radius CPG recommends performing active finger motion exercises (strength of recommendation expert opinion—“consensus”) but not to begin early wrist motion following stable fracture fixation (strength of recommendation weak/conditional—“moderate”; quality of evidence moderate—“level II”) and the use of ultrasound and/or ice as adjuvant treatment for bone healing and pain, respectively (strength of recommendation weak/conditional—“limited”; quality of evidence moderate—“level II”) [29], while the DHA radius CPG recommends use of a short-term cast or similar immobilizing bandage after insertion of a volar angular stable locking plate instead of a long-term cast (strength of recommendation weak/conditional; quality of evidence low) [30].

Regarding management of hip fracture in adults, the ANZHFR hip CPG [32] contains recommendations originating from the NICE hip CPG [31], adapted to reflect the Australian and New Zealand context. They both recommend a multidisciplinary approach to ensure that patients are mobilized on the day after surgery (NICE hip, quality of evidence low–moderate; ANZHFR hip, evidence-based recommendation grade C) and at least once a day with regular physiotherapy review, in the absence of contraindications (NICE hip, quality of evidence low to high; ANZHFR hip, consensus-based recommendation) and to provide all patients at admission with a formal, acute orthogeriatric service (NICE hip, quality of evidence low to high; ANZHFR hip, evidence-based recommendation grade B) comprising a regular assessment of patient functioning (including cognitive functions) (NICE hip, quality of evidence moderate; ANZHFR hip, practice point recommendation); early supported discharge (NICE hip, quality of evidence low to high; ANZHFR hip, evidence-based recommendation grade C); continued rehabilitation in a community hospital or residential care unit and not to exclude from rehabilitation programmes those patients admitted from care or nursing homes (NICE hip, quality of evidence low to very low) [31, 32]. AAOS hip is the only CPG included to deal specifically with management of hip fracture in the elderly population [33]. They recommend an interdisciplinary care program in patients with mild to moderate dementia (strength of recommendation strong) and supervised occupational and physical therapy across the continuum of care to improve functional outcomes and prevent falls (strength of recommendation moderate) [33].

## Discussion

We performed a search for CPGs on rehabilitation management of adults with fractures, limited to the following sites: humerus, radius, femur/hip, and tibia. High-quality CPGs including information on rehabilitation were identified for distal radius and femoral/hip fractures only [29–33]. As all the selected CPGs were primarily planned to guide orthopedic management of these fractures, specific recommendations addressing interventions for rehabilitation are largely lacking [29–33].

In patients with radius fracture treated with stable fixation or conservatively, available CPGs recommend, in all uncomplicated cases, nonsupervised exercises at home, active finger motion exercises, and use of ultrasound and/or ice [29, 30]. However, the types, frequency, and timing of these interventions are not reported, nor recommendations on appropriate functional assessment of these patients [34]. Moreover, no recommendations were provided regarding post-acute rehabilitation approaches (i.e., resistance training and fine motor and dexterity activities) needed to achieve complete recovery of autonomy in ADLs [34].

For adults with femoral/hip fracture, three CPGs were selected. The NICE hip and ANZHFR hip CPGs recommend to include a rehabilitative approach in a coordinated and multidisciplinary model of care. In this context, managing cognitive impairments and mobilizing patients from the day after surgery are the cornerstone of rehabilitation with the aim of enhancing functional recovery [31–33]. In general, the recommendations provided by the selected CPGs reflect the model of care of Western countries’ health systems, which might not be generalizable worldwide, particularly to countries with limited resources in terms of both finances and the number and skills of health professionals. In particular, available CPGs on femoral/hip fracture could be subject to some criticisms. First, although detailed orthopedic management strategies are well defined, rehabilitative interventions, in terms of type, intensity, frequency, and timing, are not adequately described and specific recommendations for the assessment of patients’ functioning are lacking. Also, regarding cognitive impairment, the selected CPGs only recommend assessment of delirium/dementia but not other emotional conditions such as depression [35].

All the CPGs for adults with fracture provide a few recommendations for rehabilitation interventions based on the Grading of Recommendations, Assessment, Development, and Evaluation (GRADE) system to define the level of evidence supporting the recommendations provided. However, a substantial issue is the overall low to very low quality of evidence in the studies, resulting in a weak/conditional strength of recommendations, on the basis of consensus of expert opinions or uncontrolled case series.

Therefore, well-designed randomized control trials and observational studies are required to enrich the evidence on rehabilitation management of adult patients with fracture.

## Conclusions

Few recommendations for rehabilitation interventions are presented, resulting in several gaps in relevant rehabilitative areas. Furthermore, the quality of the available evidence included in the CPGs is weak. There is a need for rigorous studies to inform the future development of guidelines on rehabilitation in patients with fracture to improve the state of the art and knowledge on rehabilitation for these very common health conditions. Future studies and guidelines should consider the specific situation in low- and middle-income countries.

## Data Availability

All datasets are presented in the main paper.

## Appendix 1

### Search strategy

“radius fractures” AND “rehabilitation” AND “guideline”

“distal radius fractures” AND “rehabilitation “AND “guideline”

“radius fragility fractures” AND “rehabilitation “AND “guideline”

“osteoporotic radius fractures” AND “rehabilitation “AND “guideline”

“extra articular radius fracture” AND “rehabilitation “AND “guideline”

“wedge radius fracture” AND “rehabilitation “AND “guideline”

“partial articular radius fracture” AND “rehabilitation “AND “guideline”

“sagittal radius fracture” AND “rehabilitation “AND “guideline”

“dorsal rim radius fracture” AND “rehabilitation “AND “guideline”

“volar rim radius fracture” AND “rehabilitation “AND “guideline”

“articular radius fracture” AND “rehabilitation “AND “guideline”

“metaphyseal radius fracture” AND “rehabilitation “AND “guideline”

“multifragmentary radius fracture” AND “rehabilitation “AND “guideline”

“Colles fracture” AND “rehabilitation “AND “guideline”

“Goyrand fracture” AND “rehabilitation “AND “guideline”

“Smith’s fracture” AND “rehabilitation “AND “guideline”

“Barton’s fracture” AND “rehabilitation “AND “guideline”

“Hutchinson fracture” AND “rehabilitation “AND “guideline”

“forearm fracture” AND “rehabilitation “AND “guideline”

“proximal radius fracture” AND “rehabilitation “AND “guideline”

“radial capitellum fracture” AND “rehabilitation “AND “guideline”

“radial head fracture” AND “rehabilitation “AND “guideline”

“diaphyseal radius fracture” AND “rehabilitation “AND “guideline”

“wrist fracture” AND “rehabilitation “AND “guideline”

“wrist fragility fracture” AND “rehabilitation “AND “guideline”

“Galeazzi fracture” AND “rehabilitation “AND “guideline”

“hip fracture” AND “rehabilitation “AND “guideline”

“femur fracture” AND “rehabilitation “AND “guideline”

“femoral fracture” AND “rehabilitation “AND “guideline”

“diaphyseal femur fracture” AND “rehabilitation “AND “guideline”

“diaphyseal femoral fracture” AND “rehabilitation “AND “guideline”

“femoral osteoporotic fracture” AND “rehabilitation “AND “guideline”

“fragility femoral fracture” AND “rehabilitation “AND “guideline”

“femoral head fracture” AND “rehabilitation “AND “guideline”

“femoral neck fracture” AND “rehabilitation “AND “guideline”

“subcapital femoral fracture” AND “rehabilitation “AND “guideline”

“epicondylar femur fracture” AND “rehabilitation “AND “guideline”

“transcervical fracture” AND “rehabilitation “AND “guideline”

“basicervical fracture” AND “rehabilitation “AND “guideline”

“trochanteric fracture” AND “rehabilitation “AND “guideline”

“pertrochanteric fracture” AND “rehabilitation “AND “guideline”

“intertrochanteric fracture” AND “rehabilitation “AND “guideline”

“subtrochanteric fracture” AND “rehabilitation “AND “guideline”

“femoral shaft fracture” AND “rehabilitation “AND “guideline”

“supracondylar femoral fracture” AND “rehabilitation “AND “guideline”

“complete articular distal femoral fracture” AND “rehabilitation “AND “guideline”

“partial articular distal femoral fracture” AND “rehabilitation “AND “guideline”

“extra articular distal femoral fracture” AND “rehabilitation “AND “guideline”

“distal femoral fracture” AND “rehabilitation “AND “guideline”

“tibial fracture” AND “rehabilitation “AND “guideline”

“Segond fracture” AND “rehabilitation “AND “guideline”

“toddler fracture” AND “rehabilitation “AND “guideline”

“Tillaux fracture” AND “rehabilitation “AND “guideline”

“tibial shaft fracture” AND “rehabilitation “AND “guideline”

“tibial plateau fracture” AND “rehabilitation “AND “guideline”

“tibial lateral total depression” AND “rehabilitation “AND “guideline”

“tibial medial depression” AND “rehabilitation “AND “guideline”

“tibial lateral split depression” AND “rehabilitation “AND “guideline”

“tibial medial split depression” AND “rehabilitation “AND “guideline”

“tibial oblique split depression” AND “rehabilitation “AND “guideline”

“distal tibial fracture” AND “rehabilitation “AND “guideline”

“extra articular distal tibial fracture” AND “rehabilitation “AND “guideline”

“partial articular distal tibial fracture” AND “rehabilitation “AND “guideline”

“complete articular distal tibial fracture” AND “rehabilitation “AND “guideline”

“tibial pilon fracture” AND “rehabilitation “AND “guideline”

“trimalleolar fracture” AND “rehabilitation “AND “guideline”

“bimalleolar fracture” AND “rehabilitation “AND “guideline”

“medial malleolar fracture” AND “rehabilitation “AND “guideline”

“tibial plafond fracture” AND “rehabilitation “AND “guideline”

“Destot fracture” AND “rehabilitation “AND “guideline”

“floating knee fracture” AND “rehabilitation “AND “guideline”

“diaphyseal tibial fracture” AND “rehabilitation “AND “guideline”

“humeral fracture” AND “rehabilitation “AND “guideline”

“proximal humeral fracture” AND “rehabilitation “AND “guideline”

“distal humeral fracture” AND “rehabilitation “AND “guideline”

“supracondylar humeral fracture” AND “rehabilitation “AND “guideline”

“intercondylar humeral fracture” AND “rehabilitation “AND “guideline”

“humeral greater tuberosity fracture” AND “rehabilitation “AND “guideline”

“humeral lesser tuberosity fracture” AND “rehabilitation “AND “guideline”

“humeral head fracture” AND “rehabilitation “AND “guideline”

“humeral shaft fracture” AND “rehabilitation “AND “guideline”

“humeral anatomical neck fracture” AND “rehabilitation “AND “guideline”

“humeral surgical neck fracture” AND “rehabilitation “AND “guideline”

“extra articular humeral fracture” AND “rehabilitation “AND “guideline”

“intra articular humeral fracture” AND “rehabilitation “AND “guideline”

“humeral metaphyseal fracture” AND “rehabilitation “AND “guideline”

“Holstein–Lewis fracture” AND “rehabilitation “AND “guideline”

“humeral shaft fracture” AND “rehabilitation “AND “guideline”

“humerus fracture” AND “rehabilitation “AND “guideline”

“epicondylar humeral fracture” AND “rehabilitation “AND “guideline”

“radius fractures”

“distal radius fractures”

“radius fragility fractures”

“osteoporotic radius fractures”

“extra articular radius fracture”

“wedge radius fracture”

“partial articular radius fracture”

“sagittal radius fracture”

“dorsal rim radius fracture”

“volar rim radius fracture”

“articular radius fracture”

“metaphyseal radius fracture”

“multifragmentary radius fracture”

“Colles fracture”

“Goyrand fracture”

“Smith’s fracture”

“Barton’s fracture”

“Hutchinson fracture”

“forearm fracture”

“proximal radius fracture”

“radial capitellum fracture”

“radial head fracture”

“diaphyseal radius fracture”

“wrist fracture”

“wrist fragility fracture”

“Galeazzi fracture”

“hip fracture”

“femur fracture”

“femoral fracture”

“diaphyseal femur fracture”

“diaphyseal femoral fracture”

“femoral osteoporotic fracture”

“fragility femoral fracture”

“femoral head fracture”

“femoral neck fracture”

“subcapital femoral fracture”

“epicondylar femur fracture”

“transcervical fracture”

“basicervical fracture”

“trochanteric fracture”

“pertrochanteric fracture”

“intertrochanteric fracture”

“subtrochanteric fracture”

“femoral shaft fracture”

“supracondylar femoral fracture”

“complete articular distal femoral fracture”

“partial articular distal femoral fracture”

“extra articular distal femoral fracture”

“distal femoral fracture”

“tibial fracture”

“Segond fracture”

“toddler fracture”

“Tillaux fracture”

“tibial shaft fracture”

“tibial plateau fracture”

“tibial lateral total depression”

“tibial medial depression”

“tibial lateral split depression”

“tibial medial split depression”

“tibial oblique split depression”

“distal tibial fracture”

“extra articular distal tibial fracture"

“partial articular distal tibial fracture”

“complete articular distal tibial fracture”

“tibial pilon fracture”

“trimalleolar fracture”

“bimalleolar fracture”

“medial malleolar fracture”

“tibial plafond fracture”

“Destot fracture”

“floating knee fracture”

“diaphyseal tibial fracture”

“humeral fracture”

“proximal humeral fracture”

“distal humeral fracture”

“Supracondylar humeral fracture”

“intercondylar humeral fracture”

“humeral greater tuberosity fracture”

“humeral lesser tuberosity fracture”

“humeral head fracture”

“humeral shaft fracture”

“humeral anatomical neck fracture”

“humeral surgical neck fracture”

“extra articular humeral fracture”

“intra articular humeral fracture”

“humeral metaphyseal fracture”

“Holstein–Lewis fracture”

“humeral shaft fracture”

“humerus fracture”

“epicondylar humeral fracture”
