# Correction to: Systematic review of clinical practice guidelines for adults with fractures: identification of best evidence for rehabilitation to develop the WHO’s Package of Interventions for Rehabilitation

**DOI:** 10.1186/s10195-021-00566-y

**Published:** 2021-03-01

**Authors:** Francesca Gimigliano, Sara Liguori, Antimo Moretti, Giuseppe Toro, Alexandra Rauch, Stefano Negrini, other members of the Technical Working Group, Claudio Curci, Michele Patrini, Livia Peschi, Sanaz Pournajaf, Maria Sgarbanti, Giovanni Iolascon

**Affiliations:** 1 Department of Mental and Physical Health and Preventive Medicine, University of Campania “Luigi Vanvitelli”, Largo Madonna delle Grazie n. 1, 80138 Naples, Italy; 2 Department of Medical and Surgical Specialties and Dentistry, University of Campania “Luigi Vanvitelli”, Via De Crecchio n.4, 80138 Naples, Italy; 3 Rehabilitation Programme World Health Organization, Avenue Appia 20, 1211 Geneva 27, Switzerland; 4 IRCCS Istituto Ortopedico Galeazzi, Milan, Italy; 5 Department of Biomedical, Surgical, and Dental Sciences, University La Statale, Milan, Italy

## Correction to: J Orthop Traumatol (2020) 21:20 https://doi.org/10.1186/s10195-020-00560-w

Following publication of the original article [1], the authors identified an error in the author group. Claudio Curci, Michele Patrini, Livia Peschi, Sanaz Pournajaf and Maria Sgarbanti were originally included as co-authors, but should be included as collaborators (members of the Technical Working Group).

The collaborators’ names are included in the ‘Acknowledgements’ section given below. The author group has been updated above and the original article [1] has been corrected.
